# Risk factors for coronary atherosclerotic heart disease in postmenopausal women: a meta-analysis

**DOI:** 10.3389/fcvm.2024.1434149

**Published:** 2025-01-13

**Authors:** Xin Miao, Lixing Wu, Kuiyue Wang, Yuhan Wang, Linlin Zhang

**Affiliations:** ^1^ Department of Nursing, School of Medical and Health Engineering, Changzhou University, Changzhou, Jiangsu, China; ^2^ Cardiovascular Medicine Department, Nanjing Lishui District Hospital of Traditional Chinese Medicine, Nanjing, Jiangsu, China; ^3^ Cardiovascular Medicine Department, Affiliated Hospital of Integrated Traditional Chinese and Western Medicine, Nanjing University of Chinese Medicine, Nanjing, Jiangsu, China; ^4^ The Third Clinical Medical College, Nanjing University of Chinese Medicine, Nanjing, Jiangsu, China

**Keywords:** postmenopausal women, coronary atherosclerotic heart disease, risk factors, meta-a nalysis, CHD prevention

## Abstract

**Background:**

Coronary atherosclerotic heart disease (coronary heart disease; CHD) is the leading cause of death in women worldwide, and the number of patients and deaths is increasing each year. Approximately 3.8 million women die from CHD every year globally. After menopause, estrogen levels decrease, and the risk of cardiovascular disease increases substantially; however, research on risk factors for CHD in postmenopausal women has been inconclusive.

**Objective:**

To systematically evaluate the risk factors for CHD in postmenopausal women.

**Methods:**

The PubMed, Embase, Web of Science, CINAHL, CBM, CNKI, and VIP databases were searched up to February 9, 2024, for studies on risk factors for CHD in postmenopausal women. Two researchers independently screened the literature, extracted data, and evaluated the quality of the included literature. STATA17.0 software was used for meta-analysis.

**Results:**

Nine studies involving 29,4103 patients were included. The results of the meta-analysis showed that history of six or more pregnancies (hazard ratio = 1.538, 95% confidence interval: 1.241%–1.906%) was significantly associated with risk of CHD (*P* < 0.05).

**Conclusion:**

Multiple pregnancies are associated with CHD incidence and related mortality in postmenopausal women. In the future, more and higher quality studies are needed to further verify this conclusion.

## 1 Introduction

Coronary atherosclerotic heart disease, also referred to as coronary heart disease (CHD), refers to heart disease caused by stenosis or obstruction of the vascular lumen due to coronary atherosclerosis, which leads to myocardial ischemia, hypoxia, or necrosis (1). The onset age of cardiovascular disease in women is generally around 10 years later than that in men, peaking after the menopause, which is an independent risk factor for cardiovascular disease occurrence in women. A clinical study showed that risk of death from CHD in women increased by 7-fold every 10 years after menopause (2). The 2023 edition of Chinese Menopause Management and Menopausal Hormone Therapy Guidelines (3) defines menopause as the permanent cessation of menstruation, and menopause is essentially a manifestation of ovarian failure. After menopause, estrogen levels decrease, and the risk of cardiovascular disease greatly increases. Due to the atypical clinical symptoms of CHD in postmenopausal women, for example, chest pain is not typical, only manifested as chest tightness, shortness of breath, fatigue, nausea, vomiting and other symptoms, chest pain lack of exercise related, it is easily misdiagnosed or undiagnosed (4). Further, women usually have low risk perception ability, which may lead to delayed diagnosis (5). As many factors associated with high risk of CHD are controllable, the 2018 edition of the Guidelines for the Diagnosis and Treatment of Stable Coronary Heart Disease (6) describes management measures for high-risk factors, including regulation of blood lipids, blood pressure, blood glucose in diabetic patients, physical exercise, weight management, smoking cessation, management of social and psychological factors, and alcohol management. International research on CHD in postmenopausal women has mainly focused on the identification and avoidance of risk factors before disease occurrence; however, to date, no meta-analysis has been conducted on the risk factors of coronary heart disease in postmenopausal women, and most prior studies used cross-sectional designs, which do not establish clear causality. The purpose of this study was to conduct a meta-analysis of the risk factors for CHD in postmenopausal women, to provide a basis for clinical prevention and treatment.

## 2 Materials and methods

### 2.1 Search strategy

Two researchers, MX and WKY, searched the PubMed, Embase, Web of Science, CINAHL, CBM, CNKI, and VIP databases. All cohort studies on risk factors for CHD in postmenopausal women were included. The search time limit was from the establishment of the database to February 9, 2024. The following keywords were used: postmenopause; postmenopausal period; postmenopausal; after menopause period; post-menopausal; coronary disease; coronary diseases; disease, coronary; diseases, coronary; coronary heart disease; coronary heart diseases; disease, coronary heart; Diseases, coronary heart; Heart disease, coronary; Heart diseases, coronary; Risk factors; factor, risk; risk factor; risk score*; risk factor score*; score, risk factor. Details of the search strategies are presented in Supplementary Table 1.

### 2.2 Inclusion and exclusion criteria

The criteria for inclusion of studies in the meta-analysis were as follows: (1) type of study: retrospective studies, prospective cohort study, case-control study; (2) subjects: postmenopausal women; (3) at least one exposure factor could be directly or indirectly converted into odds ratio (OR) or hazard ratio (HR) with 95% confidence interval (CI) and standard error (SE); (4) outcome: evidence for CHD diagnosis; (5) language: English.

The exclusion criteria were as follows: (1) reviews, case reports, individual cases, or non-clinical studies; (2) studies not presenting complete original data; (3) repeated publications; (4) full text report unavailable.

### 2.3 Literature screening

Two researchers, MX and WKY, independently extracted data from each study. If no agreement could be reached, another author mediated.

### 2.4 Quality evaluation and data extraction

A total of nine cohort studies were included in this study. The Newcastle-Ottawa scale (NOS) was used to evaluate the methodological quality of the studies included by the two reviewers. The level of evidence was evaluated using the JBI 2014 version of the pre-grading standard of evidence for intervention studies.

A general data table was used to extract data, including the first author, publication year, sample size, source of cases, and exposure factors and their OR, HR, and 95% CI values. All data were manually extracted by two researchers and organized into tables.

### 2.5 Data analysis

Meta-analysis was conducted using Stata 17.0 software. For combination of effect sizes, OR and HR values were selected as statistical indicators, and the corresponding 95% CI values provided. Meta-analysis models were selected to merge study data according to heterogeneity test *P* and *I*^2^ values; when *I*^2^ < 50% and *P* > 0.1, a fixed effect model was used, while a random effects model was selected when *I*^2^ ≥ 50% and *P* ≤ 0.1. Descriptive analyses of clinically understudied risk factors were conducted.

## 3 Results

### 3.1 Literature search

**Figure 1:**
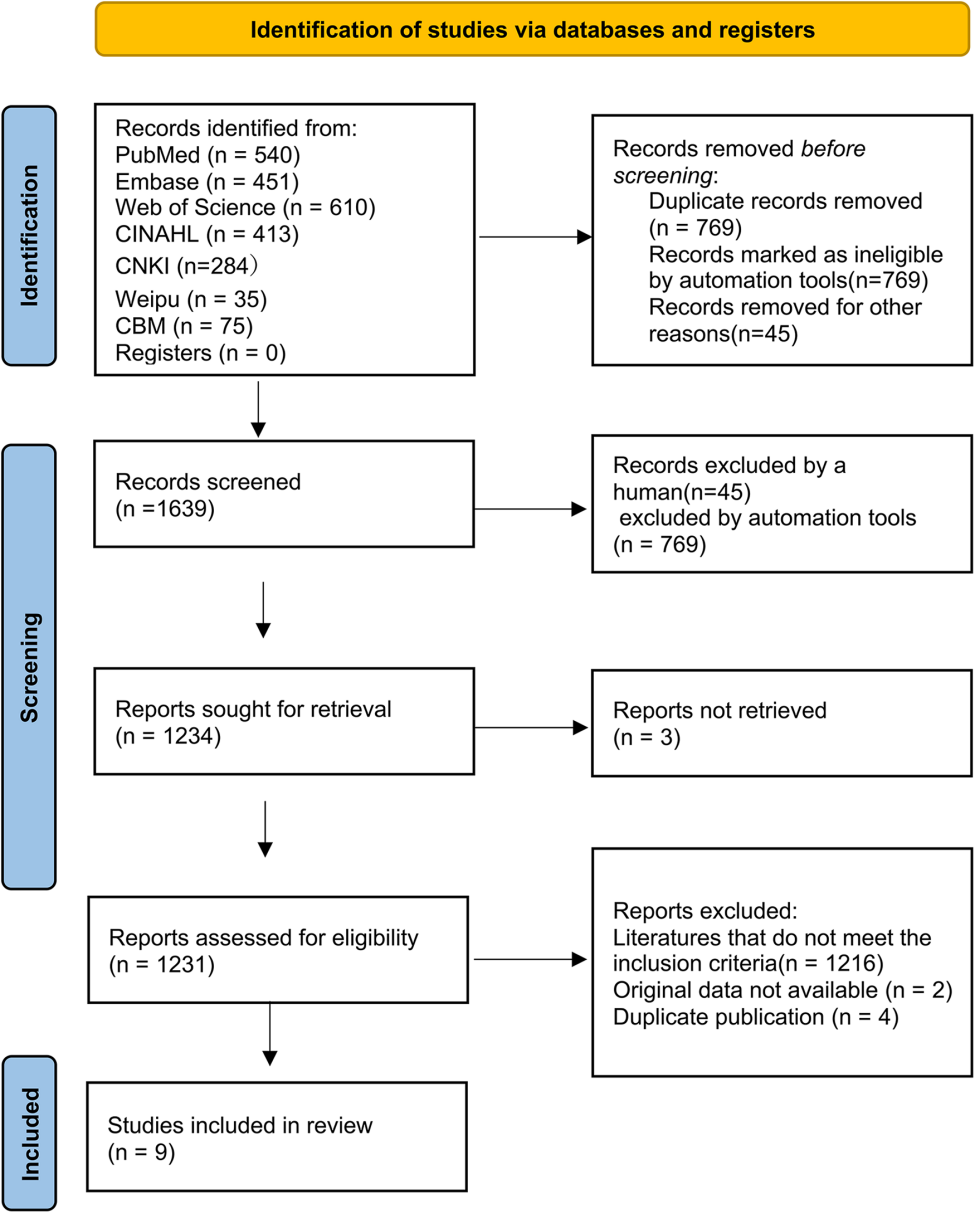
Flow diagram of search and study selection.

A total of 2,408 literature reports were obtained by searching, and 769 duplicate reports were excluded using EndNote 21 software, leading to 1,639 reports subjected to initial screening by reading the abstract, title, and results, screening layer by layer. Finally, nine reports were included for meta-synthesis (7, 15) (Figure 1). The total sample size was 294,103. The basic characteristics of the included reports are presented in Table 1. According to JBI 2014 intervention research evidence pre-grading evaluation, all nine studies were cohort studies, and their evidence levels were Level 3,3 d. Further, according to the NOS scale, the nine studies had high scores (≥ 8) (Supplementary Table 2).

**Table 1:** Basic characteristics of included studies (*n* = 9).

Study	Publication year	Country	Case source	Sample size	Variables analyzed
Everett et al. (7)	2006	USA	The Women's Health Study	15,632	Total cholesterol, LDL-C low-density lipoprotein cholesterol, high-density lipoprotein, non-HDL, High-sensitivity CRP, TC/HDL-C
Zhao et al. (8)	2018	USA	The Multi-Ethnic Study of Atherosclerosis	2,834	Total testosterone
Zhu et al. (9)	2020	Australia	25 observational studies in 10 countries	203,767	Patterns of menopause and different ages of menopause
Laughlin et al. (10)	2009	USA	Caucasian residents of southern California community	639	Total testosterone, where testosterone data were available
Cui et al. (11)	2006	Japan	The Japan Collaborative Cohort Study	37,965	Age of menopause
Ness et al. (12)	1993	USA	Framingham Heart Study; The NHEFS	4,890	Six or more pregnancies
Hashemi et al. (13)	2015	Iran	The TLGS Study	3,778	Hypertension, diabetes mellitus, family history of cardiovascular disease, total cholesterol, high-density lipoprotein cholesterol
Dam et al. (14)	2019	The Netherlands	23 centers from 10 European countries	15,402	Early menopause and surgical menopause
Yoshida et al. (15)	2021	USA	Three major USA prospective studies of CVD	9,374	Diabetes mellitus and premature menopause

### 3.2 Publication bias

Combined effect sizes for each risk factor were calculated based on a maximum of three included studies; therefore, a funnel plot was not drawn. Egger's test was used to evaluate publication bias, and indicated that publication bias was effectively controlled (*P* > 0.05).

### 3.3 Data analysis

#### 3.3.1 Risk factors for CHD in postmenopausal women

Meta-analysis of seven risk factors was conducted by combining data from the included reports. The seven risk factors subjected to meta-analysis were: multiple pregnancy history, surgical menopause, premature menopause, total testosterone, total cholesterol, high-density lipoprotein, and diabetes mellitus. Data that could not be combined for meta-analysis included information on the following potential risk factors: uric acid (16), short leukocyte telomere length (17), white blood cell count (18), long sleep duration and Women's Health Initiative Insomnia Rating Scale ≥9 points (19), frequent snoring (20), moderate to high perceived stress (21), 6-month history of panic attacks (22), history of asthma (23), total testosterone/estradiol, low-density lipoprotein C (HDL-C), non-high-density lipoprotein, high-sensitivity C-reactive protein, and total cholesterol/HDL-C (7). The combined effect size results for the seven risk factors subjected to meta-analysis are shown in Table 2.

**Table 2:** Pooled results for each risk factor.

Risk factor	Model of effect	Model of effect (95% CI)	*P* value	*χ*^2^ value
Surgical menopause	Random	1.15 (1.00, 1.32)	*P* = 0.055	4.23
Premature menopause	Random	1.07 (0.98, 1.17)	*P* = 0.13	13.59
Total testosterone	Random	1.37 (0.98, 1.92)	*P* = 0.07	2.69
Total cholesterol	Random	1.61 (0.85, 3.04)	*P* = 0.14	12.54
High density lipoprotein Cholesterol (HDL-c)	Random	0.55 (0.28, 1.07)	*P* = 0.08	12.89
Diabetes mellitus	Random	1.65 (0.79, 3.42)	*P* = 0.18	16.47

#### 3.3.2 Multiple pregnancy history as a important risk factor for CHD among postmenopausal women

Ness RB et al. (12) reported the HR values of the number of pregnancies and the risk of death from CHD based on data from the Framingham Study and the National Health and Nutrition Examination Survey I Epidemiologic Follow-up Study (NHEFS). In the Framingham Study, as compared with women who had never been pregnant, the rates of coronary heart disease adjusted for age and education level were substantially higher in women with six or more pregnancies (rate ratio,1.6; 95 percent confidence interval, 1.1–2.2). As in the Framingham Study, in the NHEFS, there was an increase in the rate of coronary heart disease adjusted for age and educational level in multigravida women (rate ratio for six or more pergnancies, 1.5; 95 percent confidence interval, 1.1–1.9).

## 4 Discussion

### 4.1 Relationship between reproductive history and CHD in postmenopausal women

The results of this meta-analysis showed that there was a causal relationship between history of multiple pregnancies and CHD in postmenopausal women, and this association was mainly seen in women who had six or more pregnancies. This result is consistent with a 2017 report based on The China Kadoorie Biobank study, where Peters (24) analyzed the correlation between the number of pregnancies and risk of cardiovascular disease in Chinese women aged 30–79 years, and found that the risk of CHD increased by 4% for each pregnancy. Further, Shuai (25) found that postmenopausal women with 1, 2, 3, and ≥4 pregnancies had 6% (OR = 1.06, 95% CI: 1.01–1.59, *P* = 0.021), 12% (OR = 1.12, 95% CI: 0.96–1.66, *P* = 0.163), 23% (OR = 1.23, 95% CI: 1.10–1.75, *P* = 0.004), and 38% (OR = 1.38, 95% CI: 1.31–3.27, *P* < 0.001), higher risks of CHD than postmenopausal women had not been pregnant, respectively, suggesting that number of pregnancies is an independent risk factor for CHD in postmenopausal women. Numerous studies have also confirmed that the number of pregnancies a women has is positively correlated with cardiovascular risk factors, including blood glucose, insulin resistance (26–29), obesity, and cholesterol levels (26, 27, 30).

The 95% CI (0.7–1.4) for the relationship between parity and CHD reported in the Nurses’ Health Study (31) included NHEFS point estimates, and two large cohort studies have also shown associations between women's reproductive history and postmenopausal CHD. Parker et al. (32) evaluated postmenopausal women from 1993 to 1998 and showed that women with a history of pregnancy loss had an increased risk of future CHD, but not of stroke. She noted that a history of pregnancy loss may be a clinically useful marker of a woman's future risk of cardiovascular disease. Wright (33) found that a history of pregnancy loss, particularly stillbirth, may be a clinically useful marker of cardiovascular disease risk in women. A recent position paper from the European Society of Cardiology explicitly referred to the apparent association between recurrent miscarriage and cardiovascular disease risk, supporting the claim that pregnancy history is an integral part of a woman's cardiovascular disease risk assessment (34). Several studies have proved that the reproductive history of a woman, including miscarriage, stillbirth, and number of pregnancies, is closely related to the risk of CHD after menopause. The American Heart Association also recommends history of pregnancy failure as a risk factor for CHD, and the 2011 revised Evidence-based Guidelines for the Prevention of Cardiovascular Disease in Women (35) called for the consideration of pregnancy complications in the assessment of lifetime risk of cardiovascular disease in women; the guidelines recommend postpartum follow-up to monitor and control cardiovascular risk factors in women with pregnancy-related complications.

Pregnancy and delivery are physiological processes for women of childbearing age. After pregnancy, a series of changes occur simultaneously in female reproductive, endocrine, cardiovascular, immune, and other systems. During pregnancy, estrogen levels are altered. Multiple pregnancies and pregnancy complications may exacerbate these changes, leading to adverse effects on the body. In addition, women who have multiple pregnancies may face greater psychological stress, social and economic burden, lack of sleep, lack of exercise, and other problems, which are related to increased risk of CHD. Gordon et al. reported that insulin resistance is a major risk factor for cardiovascular disease in perimenopausal women, and women's risk of cardiovascular disease may be affected by insulin resistance during pregnancy (36); women with multiple pregnancies have more severe insulin resistance than those with fewer pregnancies, which may be related to the changes in body fat distribution after pregnancy (37, 38, 39). Number of pregnancies as a risk factor for CHD in postmenopausal women, suggested in a study by Wright (33) that pregnancy loss, especially stillbirth, may be a clinically useful marker of cardiovascular disease risk in women. Nevertheless, a 2011 U.S. prospective study found no significant association between number of pregnancies and death from CHD (40). Thus further studies are needed to determine whether the number of pregnancies and the risk of CHD in postmenopausal women have a definite causal relationship and to explore the pathophysiological mechanisms involved.

CHD is classified as a chronic inflammatory disease, and patients with elevated levels of circulating inflammatory markers are at a higher risk for cardiovascular events. In postmenopausal women, estrogen levels decrease, diminishing the hormone's protective effects on blood vessels. This results in a state of low-grade chronic inflammation, which closely resembles the underlying mechanisms of CHD. Additionally, women often experience lipid metabolism disorders during the menopausal transition, leading to a significant increase in CHD incidence among postmenopausal women.

To effectively prevent and treat cardiovascular disease in this population, it is crucial to understand and manage risk factors while accurately identifying high-risk patients. This study found that the number of pregnancies is an independent risk factor for CHD in postmenopausal women. Therefore, preventive measures and healthcare initiatives should begin during a woman's reproductive years. It is important to enhance primary prevention strategies for CHD in women and implement effective interventions for those with high parity (six or more pregnancies).

Moreover, maternal health education and disease monitoring should be strengthened to prevent pregnancy-related complications and support postpartum recovery. We must also address the influence of family environmental factors on high-parity women, guiding them to adopt healthy lifestyles to mitigate the effects of unhealthy behaviors on their health.

### 4.2 Other risk factors for CHD in postmenopausal women

In this study, we conducted a meta-analysis on data related to the pattern of menopause, age of menopause, diabetes, total testosterone level, total cholesterol level, and high density lipoprotein cholesterol level. Although the *P* values of the combined effect sizes of these risk factors were not statistically significant, a single study demonstrated that surgical menopause and premature menopause were related to CHD incidence in postmenopausal women. Type 2 diabetes mellitus combined with early menopause may lead to a greater risk of cardiovascular disease in women. Blood lipid and hormone levels are recognized risk factors for CHD, and more relevant studies are needed to verify their roles in the pathogenesis of CHD in postmenopausal women.

The results of this study also demonstrate that multiple risk factors are associated with the incidence of cardiovascular disease in postmenopausal women, as reported in the literature. Numerous studies on blood indicators have been conducted in postmenopausal women. Sciacqua (16) and others found that uric acid levels are associated with an increased risk of cardiovascular events in postmenopausal women who do not receive hormone replacement therapy, and that uric acid is an independent predictor of cardiovascular events in postmenopausal women. Carty (17) found an association of leukocyte telomere length with CHD and mortality among Caucasian and African American postmenopausal women, while Margolis (18) found that white blood cell count >6.7 × 10^9^ cells/L can identify postmenopausal women at high-risk of CHD who cannot be identified using traditional cardiovascular disease risk factors.

Some scholars have focused on the sleep and sleep symptom of postmenopausal women. Sands (19) found that, in a fully adjusted model, postmenopausal women with high insomnia scores and long sleep duration had the highest risk of CHD, compared with postmenopausal women with medium sleep duration (HR = 1.93, 95% CI 1.06–3.51), suggesting that longer sleep duration and insomnia are associated with risk of CHD and cardiovascular disease, and may interact with one another to almost double the risk of CHD and cardiovascular disease. In addition, Sands (20) and her team found that frequent snoring during sleep was associated with risk of CHD in postmenopausal women (HR = 1.54, 95% CI 1.39–1.70). Snoring is a correlate and early symptom of obstructive sleep apnea (OSA) and may increase the risk of cardiovascular disease through atherosclerosis. Proposed biological mechanisms include that increased upper airway resistance and vibrations in the pharyngeal wall could lead to localized carotid endothelial dysfunction, a hypothesis supported by animal models (41–43). Several studies have found significant associations between heavy snoring and increased carotid intima-media thickness, plaque (44), and atherosclerosis (41), with a dose-response relationship between snoring frequency and atherosclerosis severity (41). Further research is needed to determine whether snoring alone is an independent risk factor for CVD or simply a marker for the presence of OSA, which is an established risk factor for CVD.

Other scholars focused on the psychological status of postmenopausal women. In a 3-year prospective cohort study of 10,432 postmenopausal women aged 70–75 years, Strodl (21) found that individual perceived stress remained a significant predictor of newly-diagnosed symptomatic CHD, indicating that psychosocial variables may play an important role in the new onset of CHD symptoms in older women. Further, Smoller (22) found that panic attacks are relatively common in postmenopausal women and are associated with cardiovascular morbidity and mortality in older women.

In addition, some scholars have studied the correlation between other systemic diseases and risk of CHD in postmenopausal women. For example, Marmoush (23) found that asthma was associated with a small increase in the risk of cardiovascular disease and CHD in postmenopausal women, suggesting that asthma should be actively treated to reduce the risk of cardiovascular disease in postmenopausal women.

## 5 Limitations

### 5.1 Limitations of the included literature

The literature reports included in this meta-analysis were all in English, and there may be relevant reports published in other languages that were not included. Further, the included reports could not be tested for publication bias, which would lead to instability of the results.

The evidence for a causal relationship between history of multiple pregnancies and CHD in postmenopausal women, particularly those who have had six or more pregnancies, relies on data from the same article reporting two large cohort studies (the Framingham Heart Study and the first National Health and Nutrition Examination Survey National Epidemiologic Follow-up Study). There is a lack of other large cohort studies that have investigated the association between number of pregnancies and CHD in postmenopausal women; hence, more studies are needed to strengthen and validate this conclusion.

### 5.2 Limitations of the research process

The number of studies included for analysis of each associated factor was small, and the results of this meta-analysis require further verification.

## 6 Conclusion

At present, the research on coronary heart disease in postmenopausal women has made some progress, but the pathogenesis and prevention methods are still unclear (45). Studies have shown that a woman's reproductive history (miscarriage, stillbirth, multiple pregnancies) is associated with the risk of CHD and mortality after menopause. In addition to recognized risk factors, such as blood lipid levels, premature menopause, and surgical menopause, researchers have found that other factors are related to risk of cardiovascular disease in postmenopausal women, suggesting that prevention of cardiovascular disease in postmenopausal women should start during the reproductive period; for example, by limiting numbers of pregnancies, providing appropriate healthcare during pregnancy, and conducting comprehensive evaluation of the postmenopausal population, including sleep, psychological, social, and other factors. Additional research into factors related to CHD in postmenopausal women is warranted, including exploration of the pathophysiological mechanisms involved, to inform and support evidence-based clinical practice.

## Data Availability

The original contributions presented in the study are included in the article/Supplementary Material, further inquiries can be directed to the corresponding author.
