# Supplementary material for: Risk factors for coronary atherosclerotic heart disease in postmenopausal women: a meta-analysis
Source: Front Cardiovasc Med. 2025 Jan 13;11:1434149. doi: 10.3389/fcvm.2024.1434149 (PMC11770022; doi:10.3389/fcvm.2024.1434149)
Supplement: Supplementary file 1 [file Table1.pdf]

Supplementary Table 1:

1、PubMed search strategy

STEP	search strategy
1	"Postmenopause"[MeSH Terms]
2	"postmenopausal period"[Title/Abstract]OR "period postmenopausal"[Title/Abstract] OR "Post-Menopause"[Title/Abstract] OR "Post-Menopause"[Title/Abstract] OR "post menopausal period"[Title/Abstract] OR "period post menopausal"[Title/Abstract] OR "post menopausal period"[Title/Abstract]
3	Step 1 OR Step 2
4	"Coronary Disease"[MeSH Terms]
5	"disease coronary"[Title/Abstract] OR "diseases coronary"[Title/Abstract] OR "coronary heart disease"[Title/Abstract] OR "coronary heart diseases"[Title/Abstract] OR "disease coronary heart"[Title/Abstract] OR "diseases coronary heart"[Title/Abstract] OR "heart disease coronary"[Title/Abstract] OR "heart diseases coronary"[Title/Abstract
6	Step 4 OR Step 5
7	"Risk Factors"[MeSH Terms]
8	"risk factor"[Title/Abstract] OR "health correlates"[Title/Abstract] OR "correlates health"[Title/Abstract] OR "population at risk"[Title/Abstract] OR "populations at risk"[Title/Abstract] OR "risk scores"[Title/Abstract] OR "risk score"[Title/Abstract] OR "score risk"[Title/Abstract] OR "risk factor scores"[Title/Abstract] OR "risk factor score"[Title/Abstract] OR "score risk factor"[Title/Abstract
9	Step 8 OR Step 9
10	Step 3 AND Step 6 AND Step 9

2、WOS search strategy

STEP	search strategy
1	TS=(Postmenopause)) OR TS=(Postmenopausal Period)) OR TS=(Period, Postmenopausal)) OR TS=(Post-Menopause)) OR TS=(Post Menopause)) OR TS=(Post-Menopauses)) OR TS=(Post-menopausal Period)) OR TS=(Period, Post-menopausal)) OR TS=(Post menopausal Period)
2	TS=(coronary heart disease)) OR TS=(Coronary Diseases)) OR TS=(Disease, Coronary)) OR TS=(Diseases, Coronary)) OR TS=(Coronary Heart Disease)) OR TS=(Coronary Heart Diseases)) OR TS=(Disease, Coronary Heart)) OR TS=(Diseases, Coronary Heart)) OR TS=(Heart Disease, Coronary)) OR TS=(Heart Diseases, Coronary)
3	TS=(Factor, Risk)) OR TS=(Risk Factor)) OR TS=(Health Correlates)) OR TS=(Correlates, Health)) OR TS=(Population at Risk)) OR TS=(Populations at Risk)) OR TS=(Risk Scores)) OR TS=(Risk Score)) OR TS=(Score, Risk)) OR TS=(Risk Factor Scores)) OR TS=(Risk Factor Score)) OR TS=(Score, Risk Factor)
4	Step 1 AND Step 2 AND Step 3

3、EMBASE search strategy

STEP	search strategy
1	‘Postmenopause’ OR ‘Postmenopausal Period’ OR ‘Period, Postmenopausal’ OR ‘Post-Menopause’ OR ‘Post Menopause’ OR ‘Post-Menopauses’ OR ‘Post-menopausal Period’ OR ‘Period, Post-menopausal’ OR ‘Post menopausal Period’
2	‘coronary heart disease’ OR ‘Coronary Diseases’ OR ‘Disease, Coronary’ OR ‘Diseases, Coronary’ OR ‘Coronary Heart Disease’ OR ‘Coronary Heart Diseases’ OR ‘Disease, Coronary Heart’ OR ‘Diseases, Coronary Heart’ OR ‘Heart Disease, Coronary’ OR ‘Heart Diseases, Coronary’
3	‘Factor, Risk’ OR ‘Risk Factor’ OR ‘Health Correlates’ OR ‘Correlates, Health’ OR ‘Population at Risk’ OR ‘Populations at Risk’OR ‘Risk Scores’OR ‘Risk Score’ OR ‘Score, Risk’ OR ‘Risk Factor Scores’ OR ‘Risk Factor Score’ OR ‘Score, Risk Factor’
4	Step 1 AND Step 2 AND Step 3

4、CINAHL search strategy

STEP	search strategy
1	TX：‘Postmenopause’ OR TX：‘Postmenopausal Period’ OR TX：‘Period, Postmenopausal’ OR TX：‘Post-Menopause’ OR TX：‘Post Menopause’ OR TX：‘Post-Menopauses’ OR TX：‘Post-menopausal Period’ OR TX：‘Period, Post-menopausal’ OR TX：‘Post menopausal Period’
2	TX：‘coronary heart disease’ OR TX：‘Coronary Diseases’ OR TX：‘Disease, Coronary’ OR TX：‘Diseases, Coronary’ OR TX：‘Coronary Heart Disease’ OR TX：‘Coronary Heart Diseases’ OR TX：‘Disease, Coronary Heart’ OR TX：‘Diseases, Coronary Heart’ OR TX：‘Heart Disease, Coronary’ OR TX：‘Heart Diseases, Coronary’
3	TX：‘Factor, Risk’ OR ‘Risk Factor’ OR TX：‘Health Correlates’ OR TX：‘Correlates, Health’ OR TX：‘Population at Risk’ OR TX：‘Populations at Risk’OR TX：‘Risk Scores’OR TX：‘Risk Score’ OR TX：‘Score, Risk’ OR TX：‘Risk Factor Scores’ OR TX：‘Risk Factor Score’ OR TX：‘Score, Risk Factor’
4	Step 1 AND Step 2 AND Step 3
