# Supplementary material for: Risk factors for coronary atherosclerotic heart disease in postmenopausal women: a meta-analysis
Source: Front Cardiovasc Med. 2025 Jan 13;11:1434149. doi: 10.3389/fcvm.2024.1434149 (PMC11770022; doi:10.3389/fcvm.2024.1434149)
Supplement: Supplementary file 2 [file Table2.pdf]

Supplementary Table 2:

Newcastle-Ottawa Scale for assessing the quality of studies in meta-analysis

	Brendan 2006	Di Zhao 2018	Dongshan Zhu 2020	Gail 2009	Renzhe Cui 2006	Roberta 1993	Seyed 2015	Veerle 2019	yilin 2021
**Selection**									
Representation of the exposure queue	★	★	★	★	★	★	★	★	★
Selection of non-exposed queues	★	★	★	★	★	★	★	★	★
Determination of exposure	★	★	★	★	★	★	★	★	★
No study subjects already had the disease under at the start of the study	★	★	★	★	★	★	★	★	★
**Comparability**									
Comparability of exposed and non-exposed queues	★★	★★	★★	★★	★	★★	★	★	★★
**Outcome**									
Methods of measurement of results	★	★	★	★	★	★	★	★	★

Each five-pointed star represents 1 point, and the points are accumulated at the end. Part Comparability entries can be rated up to 2 points, and the remaining entries can be rated up to 1 point each.
